# The Longevity of Mobile Apps for Cancer Recovery: Scoping Review

**DOI:** 10.2196/82448

**Published:** 2026-02-11

**Authors:** Kenneth Färnqvist, Luisa Thiele, Sophie Johnsson, Pernilla Lagergren, Anna Schandl

**Affiliations:** 1 Department of Molecular Medicine and Surgery, Karolinska Institute, Blombäcksväg 23, Stockholm, 171 77, Sweden, 46 733615317; 2 Department of Social Sciences, Technology and Arts, Luleå University of Technology, Luleå, Sweden; 3 Department of Surgery & Cancer, Imperial College London, London, United Kingdom; 4 Division of Perioperative and Intensive Care, Department of Clinical Sciences and Education, Södersjukhuset | Karolinska Institutet, Stockholm, 118 83, Sweden

**Keywords:** cancer, symptoms, neoplasm, mobile health, cancer survivorship

## Abstract

**Background:**

The number of cancer survivors is steadily increasing worldwide, leading to an increased demand for long-term follow-up and supportive care. Many survivors face ongoing physical and psychosocial issues that highlight the need for innovative management approaches. Mobile health apps offer potential benefits by facilitating patient-led follow-up, self-management, and more efficient use of health care resources. Although the market for cancer-related mobile apps has grown rapidly, their sustainability and scientific basis remain unclear. In the European Union, the Medical Device Regulation (MDR), in effect since May 2021, introduced stricter criteria for classifying medical devices, including certain software apps. While aiming to improve patient safety, the MDR could pose challenges for small companies and academic developers, potentially limiting the availability of such apps. No scoping review has delineated changes in active apps before and after implementation of the legislation regulating medical devices.

**Objective:**

This scoping review aimed to evaluate the current availability and longevity of English-language mobile apps supporting cancer recovery, with a specific focus on changes before and after the implementation of the European Union MDR, and to assess the extent to which these apps are supported by clinical evidence.

**Methods:**

Searches were conducted in mobile app stores (Apple’s App Store and Google Play) and literature databases (MEDLINE, Embase, Cochrane Library, and Web of Science), using predefined terms. Mobile apps targeting cancer recovery and published articles on their effectiveness were included. Two reviewers independently extracted data. A descriptive analysis was conducted to report trends in mobile device app availability and updates over time.

**Results:**

A total of 151 mobile apps were identified in 2018. However, by 2024, only 45 of 151 (30%) were still available. Among these, 25 of 151 (17%) were updated within the past 2 years. During the search in December 2024, 1 new mobile app supported by scientific evidence was discovered. This mobile app was developed to assist cancer survivors in managing insomnia through cognitive behavioral therapy. Rapid turnover and a potential lack of sustainability in the mobile health app market for cancer survivors were evident, with most mobile apps identified in 2018 no longer available by 2024.

**Conclusions:**

This review revealed a limited number of publicly available mobile apps that support cancer recovery. The longevity of existing mobile apps is limited, potentially because of regulatory and financial barriers. Prioritizing rigorous effectiveness trials, addressing implementation barriers, and developing sustainable business models are essential to ensure the long-term availability and success of mobile health apps in cancer survivorship care.

## Introduction

Globally, the total number of new instances of cancer is steadily rising, but treatment and survival rates are also consistently improving. Thus, the prevalence of people having undergone cancer treatments is rapidly increasing [1]. Currently, there are approximately 12 million cancer survivors in Europe [1]. Survivors often have regular health care follow-up appointments for many years, or even lifelong [2]. Hence, providing adequate cancer care is a significant challenge because of increasing demand [3]. The role of regular outpatient appointments after treatment completion has been criticized because of the lack of scientific evidence supporting their benefits and the anxiety and distress they could bring patients [4]. In contrast, some data indicate that patient-initiated follow-up appointments may be more valuable for patients and the health care system [5]. Structured clinical follow-up remains an essential component of survivorship care, with digital tools increasingly regarded as complementary rather than substitutive approaches. This complementary role highlights the need for scalable, accessible, and resource-efficient follow-up models, including mobile health apps that can enhance access to and reduce the burden on health care systems and foster patient empowerment [5,6].

While structured follow-up remains important, many patients who have completed curative-intent treatment for cancer experience incomplete recovery, with persistent physical and psychosocial problems [7]. These facts highlight the need to consider novel management strategies for cancer survivors. Use of modern communication tools, such as smartphones and engaging in patient-driven follow-up, may offer a successful way forward to improve cancer survivorship and promote more cost-effective use of health care resources [5,6]. In recent years, the market for mobile apps has grown rapidly [8], and many initiatives focusing on cancer patients have become available [9]. Nevertheless, the annual number of mobile apps created to assist individuals living with and beyond cancer does not correspond to the existing evidence of their usefulness and effectiveness [10,11]. The rapid pace of app development has outstripped the evidence base, creating a gap that researchers are now trying to address and highlight as an evaluation crisis in the field [12].

The content of mobile apps for cancer management available on the market can be divided into five main categories: (1) communicating cancer information, (2) planning and organizing cancer care, (3) interacting with others (including health care professionals and others affected by cancer), (4) enacting management strategies and adjusting to life with or beyond cancer, and (5) receiving feedback about cancer management, for example, by sharing self-monitoring reports with health care professionals [13]. For instance, almost 40 apps have been proposed to aid skin cancer detection [14]. Some apps aim to help cancer patients keep track of their treatment and therefore include functions such as schedules and treatment-related information, as well as symptom tracking. The majority of these apps have not been scientifically developed or classified as medical devices [11].

Numerous cancer mobile apps are available on the market; however, the current availability of these apps remains uncertain. The survival of an app is contingent on financial, political, and regulatory factors [15]. Apps solely used as research tools can often be sustained through research funding. Conversely, apps intended for commercial use or integration into sectors such as health care usually require ongoing external financing, which may lead to discontinuation due to struggles to secure such support. Furthermore, political decisions (eg, national reimbursement policies, procurement rules, or regulatory priorities) also influence the adoption of apps or similar software in health care, which can pose challenges for researchers [15].

The Medical Device Regulation (MDR) is a specific European Union regulation affecting the classification and approval of medical devices, including software apps [16]. The introduction of the MDR, which became applicable in May 2021, has significant implications [16]. For instance, software that provides a responsive functionality, such as offering self-care advice based on self-reported values, such as health-related quality of life was, from that date, classified as a medical device (MDR class IIa) [16]. This classification necessitates a costly and time-consuming validation process that forces these apps to comply with stringent rules for classification, technical documentation, clinical evaluation, and postmarket surveillance to remain on the market. Although the MDR has been established to enhance patient safety and create a more robust regulatory framework, it presents challenges, particularly for smaller companies and academic contexts with limited resources. The increased regulatory burden and associated costs may disproportionately affect these entities, potentially reducing the number of mHealth apps available on the European Union market [17].

No scoping review has delineated the changes in active apps before and after the implementation of the MDR. This study aimed to describe the longevity (ie, whether apps identified in 2018 remained accessible in 2024) of English-language mobile apps before and after implementation of legislation regulating medical devices by examining (1) the availability of these apps in mobile app stores and (2) the extent to which they are supported by scientific evidence.

## Methods

Our review was reported in accordance with the PRISMA-ScR (Preferred Reporting Items for Systematic Reviews and Meta-Analyses Extension for Scoping Reviews) guidelines (Checklist 1) [18]. We used the InSynQ list for items relevant to scoping reviews [19]. Quality assessment and risk of bias were not evaluated because the primary aim was to identify mobile device app measures, consistent with scoping review recommendations [18]. An internal study protocol was established before conducting the study. Because the protocol was not publicly registered, it is available from the corresponding author on reasonable request.

### Information Sources and Search for Apps

To investigate the longevity of these apps, we examined cancer-related mobile apps available since September 2018, as identified in the study by Adam et al [13], and compared them with our search conducted in December 2024. Accordingly, all mobile apps from Adam et al’s [13] study were included in separate analyses. However, different inclusion and exclusion criteria were applied to address the 2 additional research questions, and multiple approaches were used to identify available apps with supporting literature.

### Search Strategy and Screening

First, on December 2, 2024, we searched Apple’s App Store and Google Play to identify currently available cancer recovery mobile apps. We explored both the apps still available from Adam et al’s [13] study and additional apps identified through our search. Two researchers (KF and SJ) performed the searches, resolving discrepancies through discussion or consulting with a third author (AS). The search strategy described by Adam et al [13] was used. Specifically, we used the exact same keywords from the original review (“cancer,” “cancer survivor,” and “cancer survivorship”) and conducted 2 searches using an iPhone and a Samsung phone to search Apple’s App Store and Google Play, respectively.

Second, a literature search was conducted to understand which cancer recovery apps were supported by scientific evidence. Searches were conducted in MEDLINE, Embase, the Cochrane Library, and Web of Science databases. The literature search was conducted on October 4, 2022, and updated on August 15, 2024. Medical Subject Headings (MeSH) and free-text terms were identified for each search concept. The full search strategies, including all MeSH and free-text terms, are provided in Multimedia Appendix 1. For mobile apps beyond those identified in the study by Adam et al [13] but found in our December 2024 search, we explored the literature in PubMed using free-text searches for the app names. We also conducted Google searches using the names of apps identified by Adam et al [13].

We developed and pilot-tested the abstract and full-text review criteria that reflected our inclusion and exclusion criteria. For pilot testing, the reviewers assessed 10% of abstracts and full-text articles. Any discrepancies were discussed within the research group and, if necessary, the process was updated accordingly. Two researchers (KF and SJ) independently evaluated the literature search results according to the eligibility criteria using Rayyan web-based software [20]. Discrepancies were resolved through discussion or consultation with a third author (AS). Initially, titles and abstracts were screened for eligibility, followed by a full-text review of articles deemed to align with the eligibility criteria. The search strategy was developed in MEDLINE (Ovid) in collaboration with librarians at the Karolinska Institute University Library. MeSH and free-text terms were identified for each search concept. The search strategy was then translated into the other databases using the Polyglot web-based tool [21].

No language restrictions were applied to the database searches. However, studies not written in English were excluded during screening due to limited translation resources. Articles published before 2010 were excluded. The strategies were peer-reviewed by another librarian prior to execution. Deduplication was performed as described by Bramer et al [22]. A final step was added to compare the DOIs.

Third, a snowball search was conducted to check references and citations of eligible studies identified through the database searches using the Citationchaser web-based tool [23].

### Eligibility Criteria

#### Study Inclusion Criteria

As this was a scoping review, we aimed to include all relevant study designs that provided quantitative measures of impact. We considered experimental studies of any size that reported at least 1 measure of efficacy or effectiveness of a mobile app. Only studies published from 2010 onwards were eligible.

#### Study Exclusion Criteria

Observational studies, qualitative studies, editorials, reviews, opinion articles, case studies, research protocols, trial registrations, conference summaries, and papers published as abstracts were excluded. Studies that reported only qualitative measures were excluded. Due to limited resources, studies not written in English were excluded.

#### Population Inclusion Criteria

This study included primary studies of adult cancer survivors (18 years or older) who had completed treatment with curative intent before participating in the study.

#### Population Exclusion Criteria

Individuals with incurable diseases or those at the end of life were excluded. Studies that included patients younger than 18 years were excluded, even if the mean age was greater than 18 years. Studies that included patients with childhood cancer, who were subsequently included as adults, were also excluded.

#### Intervention Inclusion Criteria

We included all studies that examined smartphone apps designed for cancer survivors, with content presented in English that reported at least 1 measure of efficacy or effectiveness. The duration of the intervention had to be at least 6 weeks to ensure the intervention period was long enough to assess meaningful change in quantitative outcomes. Eligible studies required 1 treatment arm (or the treatment arm in single-arm studies) to consist solely of the mobile app or the mobile app in addition to usual care (as defined by the study authors).

#### Intervention Exclusion Criteria

We did not consider mobile apps used solely to identify or diagnose specific cancer types, provide general cancer information, manage hospital appointments or medication schedules, supply tools for health care professionals or caregivers, or support patients before or during treatment. Web-based apps were not included. Apps exclusively designed for tablets were also excluded because of their lower accessibility and use rates compared with mobile phones among the general population. Lastly, we excluded apps involving person-to-person interactions, such as cognitive behavioral therapy (CBT) or general counseling, as it would increase costs and resource demands.

#### Mobile App Characteristics Inclusion Criteria

Beyond apps identified by Adam et al [13], additional apps were eligible if they were available in one of the mobile app stores (Apple’s App Store or Google Play) and supported by evidence that met the study eligibility criteria. The eligibility of apps was evaluated using the cancer survivorship care quality framework [24], which required that the app address at least 1 of the following domains: surveillance and management of physical effects, psychosocial effects, or chronic medical conditions.

#### Mobile App Characteristics Exclusion Criteria

Apps were excluded if they did not address any survivorship domain defined in the cancer survivorship care quality framework or did not meet the availability and evidence requirements described above.

### Data Charting Process

During the study planning phase, a checklist for data extraction was developed (see Multimedia Appendix 1). We developed data extraction forms to collect relevant information from each article. The forms were piloted on 5 apps, and any discrepancies were discussed within the research group. Two researchers (KF and SJ) independently extracted relevant data (from mobile app stores and papers), except for the information on the app intervention (as described in mobile app stores), which was extracted by one reviewer (KF) and verified by another reviewer (SJ). This approach was deemed most acceptable, as the information on mobile app stores was transcribed in a free-text format and could not be extracted in precisely the same manner between reviewers. Any disagreements were resolved through discussion or by seeking input from a third reviewer (AS) when needed. The shortest follow-up was set at 6 weeks. We included all studies that investigated smartphone apps designed for cancer survivors that reported at least 1 measure of efficacy or effectiveness. We took this approach because we wanted to investigate not only existing mobile apps on the market but also apps that could potentially affect survivorship. The following information was collected from each study or the mobile app:

- Trial details (sample size, first author’s name, publication year, country, funding)
- Participant information (type of cancer)
- Mobile app specifics (name of the app, availability in app stores, latest update of the app, intervention characteristics [as described in mobile app stores], cancer survivorship care quality framework domain covered)

### Synthesis of Results

This scoping review used a narrative approach aligned with the research objectives to summarize the results. The data are presented in tabular and narrative formats, offering a comprehensive overview of the findings.

### Patient and Public Involvement

The extensive involvement of patients and their caregivers in our Surgical Care Science research partnership group [25] was integral to the study design. Their contributions encompassed proposing research questions and informing study methodologies. Feedback from these stakeholders on the study’s findings was actively solicited, and insights derived from group discussions were incorporated into the Discussion section.

## Results

### Mobile Apps Found in App Stores

In the study by Adam et al [13], 151 mobile apps were identified. Of these, 45 of 151 (30%) were still available on either Apple’s App Store or Google Play in December 2024. A total of 16 of 151 mobile apps (11%) were updated in 2024, and 25 of 151 apps (17%) were updated between 2023 and 2024. In our search in Apple’s App Store and Google Play in December 2024, we found 1 new app from 2024 that fulfilled our eligibility criteria [26]. This mobile app was developed to assist cancer survivors in managing insomnia through CBT. Detailed descriptions of all identified apps are provided in Multimedia Appendices 2-4.

In the December 2024 Google Play search, we identified 225 search results for “cancer,” 52 for “cancer survivor,” and 3 for “cancer survivorship.” In Apple’s App Store, we obtained 175 results for “cancer,” 20 for “cancer survivor,” and 10 for “cancer survivorship.” The searches for “cancer survivor” and “cancer survivorship” did not render any unique results but were covered by the initial search for “cancer” both in Google Play and in Apple’s App Store. The search did not yield any additional mobile apps. However, 1 additional mobile app was found in the literature search (Multimedia Appendix 4) [26]. The reasons for excluding apps included that they were exclusively for clinicians (n=51); were fundraising apps (n=6); were not exclusively for the treatment period (n=11); did not fulfill any of the cancer survivorship care quality framework domains (n=66); were conference apps (n=6); were for children’s cancer only (n=6); were games (n=4); were covered by Adam et al [13] (n=24); were apps for obtaining a diagnosis (n=23); were not supported by evidence (n=1); had no content related to cancer (n=164); and were not English (n=38).

### Literature Supporting Apps

**Figure 1.**
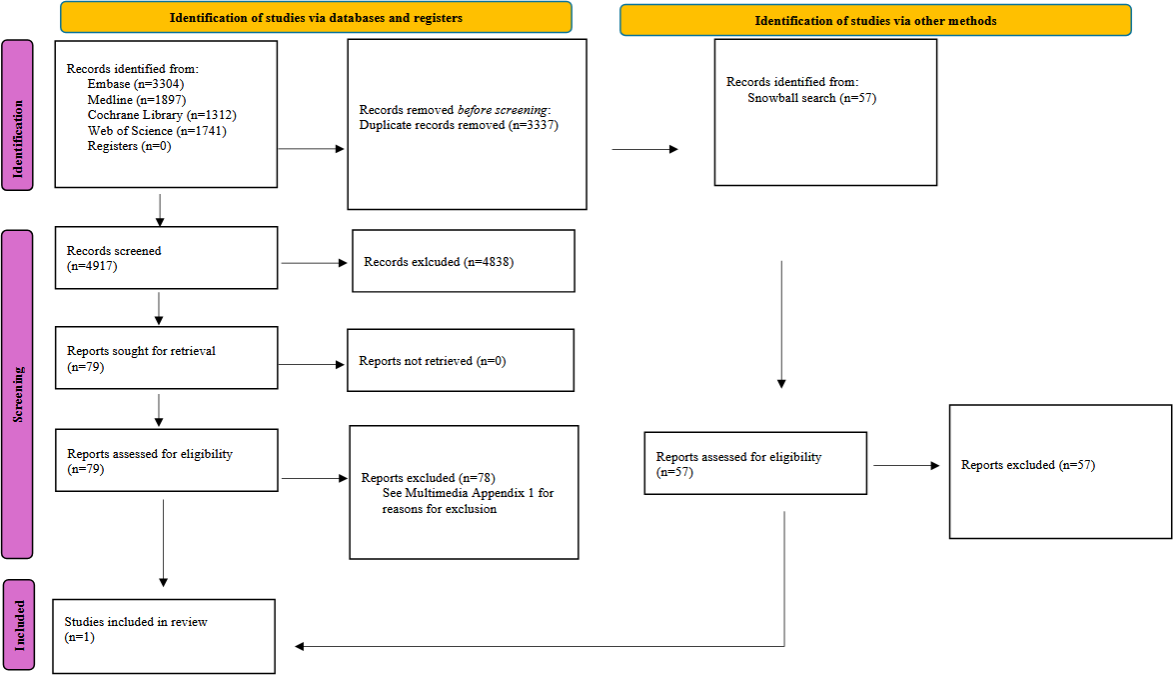
Flow diagram of search strategy (adapted from Page et al [27]).

The initial search conducted in 2022 yielded 6238 papers, and the updated search conducted in 2024 yielded 2016 new papers. A total of 3337 duplicate papers were removed. After reviewing the literature, only 1 article was included in this study. That study evaluated a mobile app developed to help cancer survivors manage insomnia through CBT. It offers a self-guided, 7-week program featuring sleep hygiene education, personalized sleep plans, relaxation techniques, and motivational interviewing. Users can track their sleep metrics and receive tailored advice to address their negative beliefs about sleep. A single-arm interventional study involving 30 participants significantly improved insomnia severity among cancer patients [26]. Figure 1 shows a PRISMA flowchart of the search strategy. See Multimedia Appendix 2 for reasons for exclusion.

## Discussion

### Principal Findings

This scoping review aimed to evaluate the durability of English-language mobile apps intended to aid cancer patients’ recovery after treatment and to determine the level of scientific evidence supporting their effectiveness. In 2018, 151 mobile apps were available, of which 45 of 151 (30%) were still available as of December 2024. Remarkably, only 16 of 151 mobile apps (11%) were updated by 2024. In addition, we found 1 new app by December 2024.

### Strengths and Limitations

To ensure the scientific rigor of this scoping review, we conducted a comprehensive literature search using 4 electronic databases, an additional snowball search to check the references and citations of eligible studies, and a broad search strategy. The review also used Apple’s App Store and Google Play, which have almost 100% coverage of the mobile app market [21]. The review followed the PRISMA guidelines for scoping reviews [18] and was methodologically robust, with an independent blinded selection of studies and data extraction (except for extraction for interventions).

However, our scoping review had some limitations. To make the review more pragmatic, we focused on mobile apps available in app stores that followed at least 1 of the cancer survivorship care quality framework domains and had supporting evidence for effectiveness without cointerventions (excluding usual care). We also excluded face-to-face interactions within the app because of the cost and implementation challenges in real-world health care settings, which have been shown to hinder mobile app survival [28]. This meant that several trials and apps were excluded. Consequently, the data used in this review do not represent the entire market. This methodology does, however, draw attention to the scarcity of cancer recovery–focused mobile apps that are available for download from mobile app stores and have some scientific backing for their efficacy.

We also conducted searches using computers and tablets to determine whether the results differed owing to the algorithms provided by the manufacturers. We noticed that the search results differed depending on who conducted the searches, when they were conducted, and the devices used for the search (eg, tablets, computers, or phones). For example, when using a web browser, the maximum number of search results for Apple’s App Store was 50, whereas for an iPhone, it was 250.

However, we consider the search terms to be accurate and representative of how a typical user searches for a mobile app related to the topic examined in this review. Finally, a limitation of searching for apps with associated studies is that the nomenclature must likely be included to identify them. Consequently, scientific support may exist for some apps, even though we did not find it.

### Implementation Challenges

Proponents of mHealth assert that its primary advantages include improved efficacy, reduced expenditure, and expeditious delivery of health care with minimal risk [29]. Nevertheless, it is not evident how digital health technologies can complement the need for in-person care interactions and health care expenses [30]. Some research findings suggest that digital interventions may contribute to increased physical activity levels. However, the outcomes regarding psychological effects remain inconclusive. Notably, the evidence supporting these findings is limited [9].

Adherence is a well-known and complex problem that can affect the outcomes of mobile app interventions [31]. Although developers have created a purportedly optimal app, its efficacy is contingent upon the health care sector’s willingness or reluctance to integrate it into existing practices [32]. Adopting new technologies in health care settings often faces challenges owing to resistance to change and concerns about workflow disruption. Health care providers may be hesitant to integrate new mobile apps into their practice if they perceive them to be time-consuming or potentially compromise patient care [9,28]. The involvement of health care professionals in the development and implementation processes is crucial for overcoming barriers. This approach ensures that the technology addresses specific requirements and integrates effectively with existing workflows [33].

The implementation of mobile apps in health care settings presents several challenges. Strict regulations, such as the MDR in Europe, have increased development costs and complexity, potentially limiting the availability of apps, particularly from smaller enterprises [16]. Financial limitations also pose a major hurdle, as sustaining high-quality health care apps requires substantial resources [17]. Additionally, technical obstacles, adoption resistance, and insufficient evidence hinder the effective integration of mobile apps into health care environments [28]. These challenges underscore the necessity for careful consideration and strategic planning to overcome these barriers and ensure successful implementation.

Most mobile apps identified in the study by Adam et al [13] are no longer available, highlighting their rapid turnover and potential lack of sustainability in the mobile health app market for cancer survivors. This high attrition rate raises concerns regarding the continuity of the research process using these digital tools, as they frequently depend on research funding [34]. Research grants may be derived from taxpayer funds, and given the research inefficiencies observed in this area, action must be taken to enhance the sustainability of these apps and avoid research waste [35]. Implementing mobile apps in health care settings would optimize the allocation of public funds instead of merely conducting pilot studies and subsequently discarding apps. Despite the emergence of new mobile apps, overall scientific evidence supporting their effectiveness remains limited [11]. Several studies suggest that apps may reduce health care use related to disease management (eg, fewer clinic visits, hospital admissions, or unplanned contacts) and the overall financial burden of the disease; however, the cost-benefit relationship remains contentious due to high development and implementation costs, as well as limited user adoption [36]. These 2 critical factors must be established when proposing a change in the care-setting environment [37,38].

### Conclusions

Despite the well-documented rapid growth of mobile health apps in the health care market, this scoping review found that there appear to be few publicly accessible, frequently updated, evidence-based apps for cancer recovery that are supported by clinical evidence. Even though many apps are available, the longevity of care recovery apps is very low. In addition, the rapid pace of mobile device app development has outstripped the evidence base, creating a gap that researchers are now trying to address while highlighting an evaluation crisis in the field. Ensuring sustained availability of mobile health apps necessitates heightened emphasis on more robust effectiveness trials, addressing barriers to implementation, and developing sustainable business models. Although some cost-effectiveness evaluations of mobile health apps exist, there is a clear need for more comprehensive and standardized assessments within care settings.

## Supplementary material

10.2196/82448 Multimedia Appendix 1 Full search strategy, checklist template, data collection process, data items, study risk of bias assessment, effect measures, mobile apps organized by last update year, and papers that were excluded after reading their full texts.

10.2196/82448 Multimedia Appendix 2 Description of available apps, according to Adam et al [13].

10.2196/82448 Multimedia Appendix 3 Longevity of cancer recovery apps, 2018-2024.

10.2196/82448 Multimedia Appendix 4 Additional cancer recovery apps found in December 2024.

10.2196/82448 Checklist 1 PRISMA-ScR checklist.

## Abbreviations

CBT: cognitive behavioral therapy
MDR: Medical Device Regulation
MeSH: Medical Subject Headings
