# Supplementary material for: The Longevity of Mobile Apps for Cancer Recovery: Scoping Review
Source: JMIR Cancer. 2026 Feb 11;12:e82448. doi: 10.2196/82448 (PMC12893644; doi:10.2196/82448)
Supplement: Multimedia Appendix 2 [file cancer-v12-e82448-s002.docx]

**Table 1.** Description of available applications, according to Adam et al 2019.

| Country | Name of app | Cancer type | Available on Apple´s App Store September 2018 | Available Android market September 2018 | Available on app store December 2024 | Available on Google Play December 2024 | Last update if available in December 2024 |
| --- | --- | --- | --- | --- | --- | --- | --- |
| U.K | 1 in 3 Cancer Support | All | Yes | Yes | No | No | N/A |
| Unclear | Adrenal cancer – others like me | All | No | Yes | No | No | N/A |
| USA | Attack cancer using hypnosis | All | Yes | Yes | No | No | N/A |
| USA | BCG Treatment | Bladder | Yes | Yes | Yes | No | 2018 |
| U.K | BECCA – Breast cancer care app | Breast | Yes | Yes | Yes | Yes | 2024 |
| USA | BELONG Beating Cancer | All | Yes | Yes | Yes | Yes | 2024 |
| Unclear | Best Prostate Cancer Treatment | Prostate | Yes | No | Yes | No | 2017 |
| Netherlands | Bible verses for cancer – strength verses | All | No | Yes | No | No | N/A |
| Singapore | BigC-Connect | All | Yes | Yes | No | No | N/A |
| USA | Bladder Cancer Manager | Bladder | Yes | No | Yes | Yes | 2024 |
| USA | Blood cancer storylines | Haematological | Yes | Yes | No | No | N/A |
| India | Blood cancer treatment | Haematological | No | Yes | No | No | N/A |
| Singapore | Boobytrapp | Breast | Yes | Yes | No | No | N/A |
| Australia | Bowel Cancer | Bowel | Yes | Yes | No | No | N/A |
| USA | BRAVE Coalition | Breast | Yes | No | No | No | N/A |
| USA | Breast Cancer Ally | Breast | Yes | No | No | No | N/A |
| Canada | Breast cancer Canada | Breast | No | Yes | No | No | N/A |
| USA | Breast Cancer Diary | Breast | Yes | No | No | No | N/A |
| USA | Breast Cancer Healthline App | Beast | Yes | No | No | No | N/A |
| USA | Breast Cancer Manager | Breast | Yes | No | Yes | No | 2023 |
| USA | Breast Cancer Social Network | Breast | Yes | Yes | No | No | N/A |
| Ireland | Breast Cancer Survivor | Breast | Yes | No | No | No | N/A |
| USA | Breast Cancer: Beyond the shock | Breast | Yes | No | No | No | N/A |
| Unclear | Breast cancer: information about breast cancer | Breast | No | Yes | No | No | N/A |
| USA | Breast friends app | Breast | Yes | Yes | No | No | N/A |
| Gibraltar | Breast Cancer Gibraltar | Breast | Yes | No | No | No | N/A |
| Unclear | Bubble health | Breast & Ovarian | Yes | Yes | No | No | N/A |
| U.K | CanAdvice+ | Breast | No | Yes | No | No | N/A |
| Unclear | Cancel Cancer | All | Yes | No | No | No | N/A |
| USA | Cancer awareness network | All | Yes | Yes | No | No | N/A |
| USA | Cancer Care and Research News | All | Yes | No | No | No | N/A |
| USA | Cancer chemotherapy and healing colours | All | Yes | Yes | No | No | N/A |
| Australia | Cancer Connect | All | Yes | No | No | No | N/A |
| Hungary | Cancer cure | All | No | Yes | No | No | N/A |
| India | Cancer curing foods | All | No | Yes | No | No | N/A |
| USA | Cancer defeated | All | Yes | Yes | No | No | N/A |
| UK | Cancer Emergency Response Tool | All | Yes | No | No | No | N/A |
| Unclear | Cancer fighting app | All | No | Yes | No | No | N/A |
| Morocco | Cancer fighting foods | All | No | Yes | No | No | N/A |
| UK | Cancer iChart | All | Yes | Yes | Yes | Yes | 2023 |
| USA | Cancer Sites@Jeff | All | Yes | No | No | No | N/A |
| USA | Cancer Support Community VVSB | All | Yes | No | No | No | N/A |
| Unclear | Cancer Surveillance | All | No | Yes | No | Yes | 2017 |
| USA | Cancer survivorship connection | All | Yes | Yes | No | No | N/A |
| France | Cancer Together | All | Yes | Yes | No | No | N/A |
| Unclear | Cancer-track and Heal | All | Yes | No | No | No | N/A |
| Unclear | Cancer Treatment Calendar | All | Yes | No | No | No | N/A |
| Unclear | Cancer treatment Tips | All | No | Yes | No | No | N/A |
| Pacific Islands | Cancer wellness | All | Yes | No | No | No | N/A |
| USA | Cancer.Fitness Community | All | Yes | Yes | No | No | N/A |
| USA | Cancer.Net mobile | All | Yes | Yes | No | No | N/A |
| Unclear | CancerAid | All | Yes | Yes | Yes | Yes | 2023 |
| USA | CancerIS | All | Yes | No | No | No | N/A |
| Australia | Cancerosity – cancer network | All | Yes | No | No | No | N/A |
| USA | CancerStop | All | No | Yes | No | No | N/A |
| Malaysia | CanDi – cancer diet app | All | No | Yes | No | No | N/A |
| Singapore | CanHOPE cancer support | All | Yes | No | No | No | N/A |
| USA | CarcinoidNETs HealthStorylines | Carcinoid | Yes | Yes | No | No | N/A |
| USA | Chemo brain | All | No | Yes | No | No | N/A |
| Unclear | Chemotherapy | All | Yes | Yes | No | No | N/A |
| USA | Cleveland Clinic Cancer Trials | All | Yes | No | No | No | N/A |
| Unclear | ClinTrial refer breast cancer | Breast | Yes | Yes | No | No | N/A |
| Unclear | ClinTrial Refer Cancer Genetics | All | Yes | Yes | No | No | N/A |
| Australia | ClinTrial Refer SA Cancer | All | Yes | Yes | No | No | N/A |
| UK | Cnected | All | Yes | No | No | No | N/A |
| USA | Colon cancer | Colorectal | Yes | Yes | No | No | N/A |
| USA | Community guide for women with cancer | All | Yes | Yes | No | No | N/A |
| USA | Don’t die 2 | All | No | Yes | No | No | N/A |
| Singapore | E-home app questionnaires | Breast | No | Yes | No | No | N/A |
| USA | Emory AWAKE | All | Yes | Yes | No | Yes | 2018 |
| USA | Eva: Cancer Support | All | Yes | Yes | No | No | N/A |
| Unclear | Eye cancer treatments | Eye | No | Yes | No | Yes | 2023 |
| India | Fight cancer naturally | All | No | Yes | No | Yes | 2022 |
| USA | Focus on lymphoma | Lymphoma | Yes | Yes | Yes | Yes | 2024 |
| Canada | For Cancer Care | All | Yes | Yes | No | No | N/A |
| USA | Hope abounds inc. | All | Yes | Yes | Yes | Yes | 2024 |
| USA | iCancerHealth Cancer Care | All | Yes | Yes | Yes | Yes | 2022 |
| USA | Inkspiration | Breast | Yes | No | Yes | No | 2015 |
| Malaysia | Inspiration of cancer survivor story | All | No | Yes | No | No | N/A |
| UK | It’s a MANTHING– Prostate Cancer | Prostate | Yes | Yes | No | No | N/A |
| India | Ketogenic therapy for cancer | All | Yes | Yes | Yes | Yes | 2022 |
| Unclear | Kidney cancer health storylines | Kidney | Yes | No | No | Yes | 2024 |
| USA | Kidney cancer manager | Kidney | Yes | No | Yes | Yes | 2024 |
| Unclear | Kids cancer meds | All | Yes | Yes | No | No | N/A |
| USA | Live like Cameron | Childhood cancers | Yes | Yes | No | No | N/A |
| USA | Liver cancer manager | Liver | Yes | No | Yes | No | 2024 |
| Unclear | Living with cancer | All | No | Yes | No | No | N/A |
| USA | Loving meditations | All | Yes | Yes | No | No | N/A |
| USA | Lung Cancer Foundation | Lung | Yes | No | No | No | N/A |
| USA | Lung Cancer Manager | Lung | Yes | No | Yes | No | 2024 |
| USA | Lung cancer navigator | Lung | Yes | Yes | No | No | N/A |
| Unclear | Lung cancer treatment | Lung | No | Yes | No | No | N/A |
| USA | Malecare prostate cancer | Prostate | Yes | No | No | No | N/A |
| USA | Markey cancer center clinical trials app | All | Yes | No | Yes | No | 2019 |
| USA | MASCC Antiemesis Tool | All | Yes | Yes | Yes | No | 2018 |
| USA | MD Anderson Mobile | All | Yes | Yes | Yes | Yes | 2024 |
| UK | Melanoma UK | Melanoma | Yes | Yes | No | No | N/A |
| Unclear | Merry medicine | All | No | Yes | No | No | N/A |
| USA | Mesothelioma Malignant Tumor chemotherapy | Mesothelioma | No | Yes | No | No | N/A |
| USA | MeTime Acupressure | All | Yes | Yes | Yes | Yes | 2020 |
| Unclear | Mindful cancer | All | Yes | No | Yes | No | 2019 |
| Unclear | Mouth cancer treatment | Oral | No | Yes | No | Yes | 2023 |
| India | MVR Cancer Centre | All | No | Yes | No | No | N/A |
| USA | My breast cancer advocate | Breast | No | Yes | No | No | N/A |
| USA | My Cancer Coach | Breast, prostate colon | Yes | Yes | No | No | N/A |
| USA | My Care Plan – cancer survivors | All | Yes | Yes | No | No | N/A |
| USA | My Head & Neck Cancer Manager | Head and neck | Yes | No | Yes | No | 2024 |
| UK | My liver | Liver | Yes | No | Yes | No | 2020 |
| UK | My Pancreas | Pancreatic | Yes | No | Yes | No | 2020 |
| USA | My Prostate Cancer Manager | Prostate | Yes | No | Yes | No | 2024 |
| USA | MyMSK | All | Yes | Yes | Yes | Yes | 2024 |
| USA | NCCN Patient Guides for Cancer | All | Yes | Yes | Yes | Yes | 2024 |
| Canada | NED for prostate cancer | Prostate | Yes | Yes | No | No | N/A |
| India | ONCompanion | All | No | Yes | No | No | N/A |
| USA | OneRemission | All | Yes | No | No | No | N/A |
| UK | Ovarian Cancer Symptoms Diary | Ovarian | Yes | Yes | No | No | N/A |
| Unclear | OWise breast cancer | Breast | Yes | Yes | Yes | Yes | 2024 |
| UK | Oxford Cancer and Haematology Outpatients | All | Yes | No | No | No | N/A |
| Unclear | Personalized sarcoma care | All | Yes | Yes | Yes | Yes | 2020 |
| USA | Phil’s friends | All | Yes | Yes | Yes | No | 2020 |
| Canada | PM Cancer Journey | All | Yes | Yes | Yes | No | 2018 |
| USA | Pocket Cancer Care Guide | All | Yes | No | No | No | N/A |
| India | Pratheeksha | All | Yes | Yes | No | No | N/A |
| Gibraltar | Prostate cancer support group Gibraltar | Prostate | Yes | No | No | No | N/A |
| Unclear | Prostate cancer treatment | Prostate | No | Yes | No | No | N/A |
| Unclear | Prostate cancer we have your back | Prostate | Yes | No | No | No | N/A |
| USA | Qigong for cancer healing and prevention | All | Yes | Yes | Yes | Yes | 2023 |
| Canada | Radiotherapy | All | No | Yes | No | No | N/A |
| Germany | RB-World App | Retinoblastoma | Yes | Yes | Yes | Yes | 2024 |
| Unclear | Safe and easy cancer/ Easy ways to treat cancer | All | No | Yes | No | No | N/A |
| USA | SCICancer Clinical Trials | All | Yes | Yes | Yes | No | 2021 |
| USA | Self Care During Cancer | All | Yes | Yes | No | No | N/A |
| USA | Signs and symptoms breast cancer | Breast | Yes | Yes | No | No | N/A |
| USA | Skin Cancer Manager | Skin | Yes | No | Yes | Yes | 2024 |
| USA | Stupid Cancer | All | Yes | Yes | No | No | N/A |
| Unclear | Superfood to fight for cancer | All | No | Yes | No | No | N/A |
| Netherlands | Survivor care | All | Yes | No | Yes | No | 2023 |
| Unclear | SwiSupport – HealingMusic | All | Yes | No | No | No | N/A |
| USA | T.I.N.A | All | Yes | Yes | Yes | Yes | 2022 |
| Unclear | Testicular cancer | Testicular | No | Yes | No | No | N/A |
| Unclear | Thrivor | All | Yes | Yes | No | No | N/A |
| Unclear | Treat prostate cancer | Prostate | No | Yes | No | No | N/A |
| Unclear | Treating bladder cancer | Bladder | No | Yes | No | No | N/A |
| USA | Triple negative breast cancer | Breast | Yes | Yes | Yes | Yes | 2023 |
| Unclear | Twist out cancer | All | Yes | No | No | No | N/A |
| Unclear | Types of cancer treatment | All | No | Yes | No | No | N/A |
| Netherlands | Untire: Beating cancer fatigue | All | Yes | Yes | Yes | Yes | 2023 |
| Unclear | Various cancer cures | All | No | Yes | No | No | N/A |
| Unclear | Ways to fight off cancer | All | Yes | Yes | No | No | N/A |
| USA | Whip Cancer | All | Yes | No | No | No | N/A |
| Spain | Yoga vs. Cancer | All | No | Yes | No | No | N/A |

Adam R, Mcmichael D, Powell D, Murchie P. Publicly available apps for cancer survivors: a scoping review. BMJ Open. 2019;9(9):e03251
