# Supplementary material for: The Longevity of Mobile Apps for Cancer Recovery: Scoping Review
Source: JMIR Cancer. 2026 Feb 11;12:e82448. doi: 10.2196/82448 (PMC12893644; doi:10.2196/82448)
Supplement: Multimedia Appendix 3 [file cancer-v12-e82448-s003.docx]

**Table 2.** Longevity of cancer recovery applications: Description of applications available from 2018-2024

| **Study** | **App** | **Patients (n)** | **Intervention** |
| --- | --- | --- | --- |
| No studies found | BCG Treatment | N/A | This app offers easy-to-understand and reliable resources for self-education. It is designed to assist patients in handling treatment side effects and minimising non-compliance risk. Specifically tailored for bladder cancer patients undergoing BCG therapy, the app contains interactive tools that are visually engaging and have been developed by a diverse team of experts. The app aims to empower patients by enhancing their understanding and reducing psychological distress. The content is written at a sixth-grade reading level for accessibility. |
| No studies found | BECCA | N/A | The app provides daily, carefully selected tips for post-breast cancer recovery, including advice on diet, exercise, and mental well-being. Users can save and customise content relevant to their recovery journey. The app is designed to help users confidently resume everyday life. |
| No studies found | BELONG Beating Cancer | N/A | The app is a free, anonymous mobile platform designed to support cancer patients and caregivers. Key features include support groups tailored to different cancer types, the ability to connect with other patients and healthcare professionals, and tools such as a medical binder and a clinical trial matching service. |
| No studies found | Best Prostate Cancer Treatment | N/A | The app provides information on natural treatments, including whole-leaf Aloe vera for various cancer types, such as pancreatic and breast cancer. It offers clinical insights on managing symptoms and discusses the potential benefits for various cancers. The app also fosters community engagement through social media platforms like Instagram, Twitter, and Facebook, allowing users to stay connected and participate in real-time conversations. Additionally, the app features a YouTube channel with videos on cancer treatment, industry facts, and more. |
| No studies found | Bladder Cancer Manager | N/A | The app helps users to manage symptoms, medications, and treatments associated with bladder cancer. It allows users to track their progress through daily journals, which generate easy-to-read charts to identify trends and areas of concern. The app integrates with the Health app, enabling users with heart conditions to send heart rate data to their doctors if they wish to do so. Features include medication reminders, photo uploads to share symptoms with care providers, and access to the latest bladder cancer information. |
| No studies found | Breast Cancer Manager | N/A | The app helps users to manage their daily breast cancer symptoms, medications, and treatments. It integrates with the Health app, allowing users with heart conditions to send heart rate data to their doctors while ensuring that no health data is shared without the user's consent. The app enables users to track their progress through a daily journal and displays the data in colourful charts to identify trends and areas of concern. Its features include medication reminders, the ability to upload photos to share symptoms with care providers, and access to the latest breast cancer information. |
| No studies found | Cancer iChart | N/A | The app is designed to assist healthcare professionals and cancer patients check for potential interactions between anti-cancer drugs and other medications. It uses a "Traffic Light" system (red, amber, yellow, green) to provide recommendations, along with a summary of each interaction and the quality of evidence (ranging from very low to high). |
| No studies found | CancerAid | N/A | The app is designed to help cancer patients manage the physical and psychological side effects of their diagnosis, treatment, and recovery. The app aims to empower users by providing tools to organize their care and reduce feelings of disorganisation, confusion, isolation, and overwhelm. It supports patients throughout their cancer journey, helping them regain control over their health. |
| No studies found | Cancer Surveillance | N/A | The app helps you monitor cancer progress for yourself or others by organising appointments, tests, and results. You can track everything from biopsies, MRIs, and blood tests to symptoms, treatments, and surgery dates. It is suitable for all types of cancer and includes a predefined list of tests, markers, and events, with options to add more. |
| No studies found | Emory AWAKE | N/A | The app is a positive psychology program focusing on goal-oriented thinking for cancer survivors |
| No studies found | Eye cancer treatments | N/A | The app lets you need to know about eye cancer. Learn to recognise symptoms, discover treatment options, manage emotional stress, and cope with side effects. It features informative articles, helpful resources, and valuable tools and is your guide to understanding and managing eye cancer. Key features include offline reading, multilingual support, and updates on the latest research. |
| No studies found | Fight cancer naturally | N/A | The app offers a holistic perspective that involves natural food, herbs, a keto diet, and natural supplements. |
| No studies found | Focus on Lymphoma | N/A | The Focus on Lymphoma app offers patients and caregivers tools to manage lymphoma and access up-to-date resources from the Lymphoma Research Foundation. It provides disease-specific information, including trackers for medications, blood counts, and symptoms. The app also helps users manage doctor appointments, record sessions, and organise questions for their healthcare providers. Additionally, it connects users to support services like clinical trial searches, peer support, and financial assistance. The app also includes resources on preparing for CAR T-cell therapy. |
| No studies found | Hope abounds cancer network | N/A | The app lets users access educational articles, online support forms, and details about upcoming events. The app also features interviews with medical experts and cancer survivors, providing encouragement and guidance. |
| No studies found | iCancerHealth Cancer Care | N/A | The app lets cancer patients connect virtually with their care professionals between visits, offering a personalised experience tailored to individual needs. Patients can send secure messages, report symptoms, track medications, and receive reminders. The app provides access to valuable educational resources, facilitating comprehensive support throughout the cancer journey. |
| No studies found | Inkspiration | N/A | The app offers breast cancer survivors a virtual tool to explore designs for mastectomy tattoos. Users can upload personal photos or select a body type to visualise potential tattoos, which can help their decision-making process. The app includes a library of designs and also assists in connecting users with professional tattoo artists who have experience in creating mastectomy tattoos. |
| No studies found | Ketogenic therapy for cancer | N/A | The app helps users manage cancer therapy using a ketogenic diet, natural supplements, and herbs. Plan meals, monitor your glucose-ketone index (GKI), and analyse therapeutic ketosis to stay on track. Key features include personalised diet plans, GKI analysis, and progress tracking. This resource is ideal for both doctors and patients who are utilising a ketogenic approach for cancer care. |
| No studies found | Kidney cancer health storylines | N/A | The app allows kidney cancer or renal cell carcinoma users to monitor symptoms, fatigue, medications, and moods. It provides personalised health management tools such as a symptom and fatigue tracker, medication reminders, and mood journaling. Users can synchronise data from other health apps to enhance communication with healthcare providers and better understand their health status between appointments. |
| No studies found | Kidney cancer manager | N/A | The app assists users in managing kidney cancer symptoms and overall health by tracking daily activities, medications, and visible symptoms through a journal. It integrates with the Health app to share heart rate data with healthcare providers and visualises health trends using pie charts. Users can connect with providers, access resources related to kidney cancer, and enjoy gamified features such as earning badges. The app also supports the management of multiple conditions, offers reminders and educational tools, and provides guided in-app assistance for a seamless user experience. |
| No studies found | Liver cancer manager | N/A | The app helps users to manage their daily liver cancer symptoms, medications, and treatments. It integrates with the Health app, allowing users with heart conditions to send heart rate data to their doctors while ensuring that no health data is shared without the user's consent. The app enables users to track their progress through a daily journal and displays the data in colourful charts to identify trends and areas of concern. |
| No studies found | Lung Cancer Manager | N/A | The app helps users to manage their daily lung cancer symptoms, medications, and treatments. It integrates with the Health app, allowing users with heart conditions to send heart rate data to their doctors while ensuring that no health data is shared without the user's consent. The app enables users to track their progress through a daily journal and displays the data in colourful charts to identify trends and areas of concern. |
| No studies found | Markey cancer center clinical trials app | N/A | The app provides easy access to information about ongoing clinical trials. It enables patients and researchers to search, view, and save detailed information on clinical trials and share it with others. |
| No studies found | MASCC Antiemesis Tool | N/A | The app helps cancer patients undergoing chemotherapy by enabling them to track and report the severity of nausea and vomiting. It utilises a validated questionnaire created by the Multinational Association for Supportive Care in Cancer (MASCC) to evaluate these side effects over two timeframes: within the first 24 hours and from day two to four after chemotherapy. Patients can send real-time results to their healthcare providers for prompt symptom management. |
| No studies found | MD Anderson Mobile | N/A | The app offers MD Anderson Cancer Center patients access to their personal health records, appointment management, secure messaging with healthcare teams, and educational resources. Users can complete pre-visit questionnaires, view balances, and make payments. Additionally, the app provides features such as directions to appointments, shuttle schedules, and access to news and social media, enhancing patient support and engagement. |
| No studies found | MeTime Acupressure | N/A | The app teaches cancer survivors how to perform self-acupressure to reduce fatigue. |
| No studies found | Mindful cancer | N/A | The app provides daily mindfulness and meditation sessions to help cancer patients during their diagnosis, treatment, and recovery. Users can participate in guided meditations to reduce stress, improve sleep, manage anxiety, and enhance emotional well-being. The sessions are customised for various stages of the cancer journey to support mental and physical healing. |
| No studies found | Mouth cancer treatment | N/A | The app offers a complete guide to oral cancer, including information on early signs, survival rates, and available treatments. Its goal is to educate users about the essential aspects of oral cancer, helping them stay informed and take proactive steps for their health. |
| No studies found | My Head & Neck Cancer Manager | N/A | The app assists patients in managing head and neck cancer symptoms by allowing them to track pain, moods, medications, and treatments through a daily journal. It then transforms this data into charts for easy trend analysis and provides medication reminders. Users can share visible symptoms with healthcare providers, who can monitor progress remotely. The app also offers educational resources and integrates with health data from Apple devices for enhanced condition management. |
| No studies found | My liver | N/A | The app is designed for NHS patients diagnosed with liver cancer. It aims to guide them through their treatment journey and features five main sections: My Liver, My Tests, My Treatment, Help & Information, and My Notes. The My Liver section provides an overview of liver anatomy, functions, and potential issues. In the My Tests section, patients can find information on diagnostic procedures—the My Treatment section details possible treatments such as chemotherapy and surgery. Patients can access patient support resources, including care services, financial assistance, and helpful websites in the Help & Information section. Lastly, the My Notes section allows patients to record questions for healthcare appointments or select from pre-written questions. The app aims to empower patients by providing essential information and support. |
| No studies found | My Pancreas | N/A | The app is designed for NHS patients diagnosed with pancreatic cancer. It aims to guide them through their treatment journey and features five main sections: My Pancreas, My Tests, My Treatment, Help & Information, and My Notes. The My Pancreas section overviews pancreas anatomy, functions, and potential issues. In the My Tests section, patients can find information on diagnostic procedures—the My Treatment section details possible treatments such as chemotherapy and surgery. Patients can access patient support resources, including care services, financial assistance, and helpful websites in the Help & Information section. Lastly, the My Notes section allows patients to record questions for healthcare appointments or select from pre-written questions. The app aims to empower patients by providing essential information and support. |
| No studies found | My Prostate Cancer Manager | N/A | The app helps patients manage prostate cancer by tracking daily symptoms, pain, medications, and treatment progress through a user-friendly journal. The data is transformed into visual charts. It includes features for medication reminders, uploading photos of visible symptoms, and connecting with healthcare providers for remote monitoring. Users can access the latest information on prostate cancer and use in-app help for guidance. The app also integrates with the Health app for heart condition management, allowing users to control data sharing with their doctors. |
| No studies found | MyMSK | N/A | The patient app is designed for individuals receiving care at Memorial Sloan Kettering Cancer Center. It enables patients and caregivers to securely access their medical information, view and share test results, manage appointments, and save details to their mobile calendars. Additionally, the app supports telemedicine visits, messaging with care teams, requesting prescription refills, completing health questionnaires, reading educational materials, and viewing and paying bills. |
| No studies found | NCCN Patient Guides for Cancer | N/A | The app offers user-friendly resources based on the clinical practice guidelines healthcare professionals use to identify the best treatment options for cancer patients. These guidelines outline alternative cancer treatments likely to produce the best results. The app includes a list of questions to ask your doctor, easy-to-understand illustrations, and comprehensive glossaries of terms used in cancer care. |
| No studies found | OWise breast cancer | N/A | The app is specifically designed to assist individuals in regaining control after receiving a breast cancer diagnosis. It provides personalised, reliable information and practical support in one accessible platform. Users can track over 30 side effects from treatments such as hormone therapy, chemotherapy, and immunotherapy, eliminating the need for traditional paper diaries. The app offers personalised insights by generating tailored reports based on the user's diagnosis, allowing them to track symptoms and create a list of questions for their doctor. Additionally, it provides a centralised platform for users to keep an overview of their treatment plan, track appointments, and record conversations with their healthcare team. Users can also securely store private notes and photos. Furthermore, the app enables improved communication by allowing users to share tracked symptoms with their care team and access credible, evidence-based content to enhance discussions with their doctor. |
| No studies found | Personalized sarcoma care | N/A | The app informs users about soft tissue sarcoma, a rare cancer affecting muscles, nerves, fat, and other soft tissues. The app is designed for patients with grade III sarcoma undergoing surgery and radiotherapy. It estimates outcomes such as survival and recurrence based on individual patient and tumour data. |
| No studies found | Phil’s friends | N/A | The app has content and resources to help support cancer survivors and stay connected to the organisation. Cancer is strong, but hope is stronger. With this app, you can request a Care Package to be sent anonymously to someone you know who is affected by cancer, sign up to receive consistent support through our Cards of Hope program, and submit prayer requests for others to pray for you. |
| No studies found | PM Cancer Journey | N/A | The application is designed to assist cancer patients, their families, and friends to feel more in control after a diagnosis. It offers essential information and services related to treatment at the Princess Margaret Cancer Centre, ensuring users can access reliable health information. Features include a guidebook with articles offering helpful tips throughout the cancer journey, a questions tracker to keep a list of questions for the healthcare team, a doctor directory to search for contact information for doctors at Princess Margaret, a notes section to record and store important information, brochures providing reliable health information created by clinicians and healthcare professionals, access to support services at Princess Margaret and in the community, a list of trusted websites offering credible health information, and the ability to create shortcuts to access important pages easily. |
| No studies found | Qigong for cancer healing and prevention | N/A | The app allows users to stream or download healing qigong exercises. Features include natural healing through qigong, user-friendly instructions that can be followed while sitting or standing, and side-to-side mirror-view demonstrations for better guidance. The app aims to support individuals in their healing journey through the practice of qigong. |
| No studies found | RB-World App | N/A | The app allows the user to step into the diverse community of individuals worldwide affected by retinoblastoma, including survivors, parents, doctors, ophthalmologists, and more. Experience inspiring stories, insights, medical advice, and friendships just around the corner. |
| No studies found | SCICancer Clinical Trials | N/A | This app provides access to over 250 actively recruiting cancer clinical trials at the Stanford Cancer Institute, led by renowned physicians across various treatment areas. Users can browse trials by cancer conditions or enter keywords for specific searches. Additionally, they can send trial details directly to their email account and contact individual trial study coordinators or the Cancer Clinical Trials Office through the app. The app aims to make valuable clinical trial information easily accessible for patients and caregivers seeking cancer treatment options. |
| No studies found | Skin Cancer Manager | N/A | The app helps individuals manage skin cancer by tracking symptoms and treatments. It integrates with the Health app, allowing users to manage heart-related data and share it with their doctors while maintaining control over their health information.  Features include a daily journal to track progress and symptoms using sliders and buttons, with data in easy-to-read charts. It also offers medication management to keep track of medications and treatments, including reminders and allows users to share images of visible symptoms with healthcare providers. The app also provides connectivity with care providers for ongoing monitoring and support, access to up-to-date resources on skin cancer in the "Learn More" section, and in-app help to guide users through the app's features for optimal use. The Skin Cancer Manager aims to empower users by providing tools and resources to help them take control of their health journey. |
| No studies found | Survivor care | N/A | The app is designed to support cancer survivors during their post-treatment phase. It focuses on managing treatment after-effects and facilitating early detection of recurrence. The app offers resources for recovery and maintaining overall health. It also provides interactive tools for tracking questions for healthcare providers and recording answers. Additionally, it allows users to illustrate questions and responses with pictures. The app's key features include a personalised care plan that can read QR codes generated by specialists. These codes encode tailored survivorship care plans for individuals, particularly benefiting testicular cancer survivors. It also provides a care plan overview, a comprehensive summary of treatment received and follow-up plans to help survivors and their caregivers navigate the survivorship phase. Furthermore, users can manage to-dos and appointments with caregivers, including setting reminders. |
| No studies found | T.I.N.A | N/A | The app educates patients and caregivers on preventing infections during chemotherapy. For Cancer Patients, the app offers interactive learning to reduce the risk of infection during chemotherapy. It also provides information on what to do if signs of infection are noticed. Patients can take on the role of a virtual patient and interact with Tina, a virtual oncologist, to get their questions about neutropenia and infection prevention answered. The app allows caregivers to practice sharing information about neutropenia with patients through interactive conversations. Caregivers can step into the role of Tina, the virtual oncologist, and learn how to discuss this topic effectively with cancer patients. They can also interact with a virtual patient to practice a realistic conversation. |
| No studies found | Triple negative breast cancer | N/A | The app allows individuals diagnosed with triple-negative breast cancer or their loved ones to engage in a conversation with Linda, a virtual coach. It provides a safe environment for users to ask questions and explore topics relevant to their situation. This app empowers users to explore their thoughts and feelings at their own pace while providing essential information and support. Key features include personalised interaction, informative content, and resource access. |
| No studies found | Untire: Beating cancer fatigue | N/A | The app includes education, lifestyle tips, mind and body exercises, community support, and weekly reporting. It helps manage energy levels, adjust behaviours, and identify patterns. It's effective for all individuals, regardless of cancer diagnosis. The program covers fatigue, anxiety, boundaries, sleep, self-care, and nutrition. |
