# Supplementary material for: The Longevity of Mobile Apps for Cancer Recovery: Scoping Review
Source: JMIR Cancer. 2026 Feb 11;12:e82448. doi: 10.2196/82448 (PMC12893644; doi:10.2196/82448)
Supplement: Multimedia Appendix 4 [file cancer-v12-e82448-s004.docx]

**Table 3.** Additional cancer recovery application(s) found in December 2024

| **Country** | **Study** | **App** | **Type of cancer** | **Patients (n)** | **Available on Google Play** | **Available on app store** | **Funding** | **Cancer survivorship care quality framework domain** | **Intervention** |
| --- | --- | --- | --- | --- | --- | --- | --- | --- | --- |
| USA | Arring et al. 2024 | MI sleep coach | Breast, colon, prostate | 30 | Yes | Yes | The work was supported by the Breast Cancer Research Foundation and by the National Cancer Institute (P30CA046592) by the use of the Rogel Cancer Center Health Communications Shared Resource | Surveillance &  management of  psychosocial  effects | This app was developed to assist cancer survivors in managing insomnia through cognitive behavioural therapy for insomnia. To encourage adherence, it offers a seven-week, self-guided program with features such as sleep hygiene education, personalised sleep plans, relaxation techniques, and motivational interviewing (MI). Users can track their sleep metrics and receive tailored advice to address negative beliefs about sleep. Additionally, the app provides just-in-time behavioural prompts throughout the day. The program incorporates a digital Sleep Coach avatar for engaging and supportive conversations and a points-based reward system for positive behaviour changes. |

Arring N, Barton DL, Lafferty C, Cox B, Conroy DA, An L. Mi Sleep Coach Mobile App to Address Insomnia Symptoms Among Cancer Survivors: Single-Arm Feasibility Study. JMIR Formative Research. 2024;8:e55402.
